# Evaluations of 3D-Printed Surgical Mask Tension Release Bands for Common COVID-19 Use with Biomechanics, Sensor Array System, and Finite Element Analysis

**DOI:** 10.3390/s22155897

**Published:** 2022-08-07

**Authors:** Kuo-Chih Su, Chun-Hsiang Wang, Yu-Chun Yen

**Affiliations:** 1 Department of Medical Research, Taichung Veterans General Hospital, Taichung 407, Taiwan; 2 Department of Biomedical Engineering, HungKuang University, Taichung 433, Taiwan; 3 Department of Chemical and Materials Engineering, Tunghai University, Taichung 407, Taiwan

**Keywords:** mask, 3D printing, surgical mask tension release band, sensor array system, biomechanics, finite element analysis

## Abstract

A mask is one of the most basic protections to prevent the transmission of COVID-19. Surgical mask tension release bands (SMTRBs) are commonly used to ease the pain caused by prolonged mask use. However, the structural strength of SMTRBs and the effect that wearing masks with SMTRBs has on the face are unclear. Thus, this study assessed the mechanics of seven different types of 3D-printed SMTRBs. In this study, a tensile testing machine, a sensor array system, and finite element analysis were used to evaluate the mechanisms of seven SMTRBs. The tensile testing machine was applied to measure the breaking strength, elongation, stiffness, and rupture of the band. The sensor array system was used to calculate the pressure on the face when the band was used together with the mask. Finite element analysis was applied to evaluate the level of stress on the SMTRB structure when each of the seven bands was subjected to external force. The results demonstrated that thick SMTRBs put more pressure on the face but had greater structural strength. The thinner bands did not break easily; however, the mask ear loops tended to slip off more often. In addition, the size of the band hook affected the magnitude of the external force. This study provides a biomechanical reference for the future design of SMTRBs.

## 1. Introduction

Recently, COVID-19 has been a topic of utmost concern worldwide. According to the World Health Organization website [1], about 571 million people have been infected by COVID-19, with 6.3 million deaths (data accessed on 29 July 2022). The COVID-19 virus is transmitted via airborne microdroplets (also known as aerosols) [2,3,4,5]. To prevent the droplet and contact transmission of COVID-19, self-protection has become increasingly important [6]. Masks are basic protective devices used by medical staff and the general public to prevent COVID-19 infection [7]. Common mask designs include those held in place by elastic ear loops that wrap around both ears. This produces constant pressure and friction against the skin, leading to a sensation of pain for the wearer. Mask discomfort due to heat, pressure, and pain also increases after prolonged use [8], and such discomfort may affect an individual’s compliance and willingness to wear a mask [9].

To overcome the problems of face fit and pressure discomfort behind the ear, surgical mask tension release bands (SMTRBs) are employed to relieve pain [10]. During the current pandemic, because of the need to produce such bands quickly and in large quantities, the most common production method is 3D printing [11,12]. The advantages of 3D-printing technology include the customization of object design, mass production, and cheap materials. Hence, a large amount of other medical equipment has also been produced by 3D printing during the pandemic [13,14,15]. The NIH 3D Print Exchange website [16] provides several styles of SMTRB (also referred to in this study as the band), which saves the time necessary to generate a design model for such a band. In addition, 3D-printing technology allows complex designs to be used in such bands due to the lack of geometrical restrictions on the physical shapes of the to-be-printed objects. The design of the band is modified to reduce pressure behind the ear, reduce discomfort, and increase mask durability [17]. The quality of the design may also enhance the function and service life of the band. However, there is no current study offering a mechanical evaluation of SMTRB designs; as such, this study evaluates different SMTRB designs using mechanical evaluations.

To assess the biomechanics of SMTRBs, a structural mechanics evaluation was performed for bands with different designs. Mechanical testing is a common method for such structural evaluations. Some researchers have used mechanical testing to evaluate mask materials [18]. Since the pressure exerted by masks on the face causes discomfort to the wearer, pressure on the face during mask-wearing was used as a basis for evaluation. Previous studies used sensor array systems to measure the pressure distribution on the human body [19]. Therefore, in this study, we used a sensor array system to measure the external force and pressure distribution on the face. In addition, finite element analysis is also a common method for the mechanical evaluation of the design of medical equipment [20]. Therefore, finite element analysis is a very suitable way to evaluate the structural design of SMTRBs. Here, the results of a finite element analysis were used to evaluate structural designs and to make informed recommendations for improvement and optimization.

According to the literature, an SMTRB is a pain-relief device. The design of such a band may affect its function and lifespan. In brief, the main purpose of our study is to use a tensile testing machine, a sensor array, and finite element analysis to evaluate the structural strength of different styles of 3D-printed SMTRBs, as well as their pressure distribution patterns on the face during mask-wearing. Our findings provide a biomechanical reference for the future design of SMTRBs.

## 2. Materials and Methods

### 2.1. SMTRBs of Different Styles

**Figure 1:**
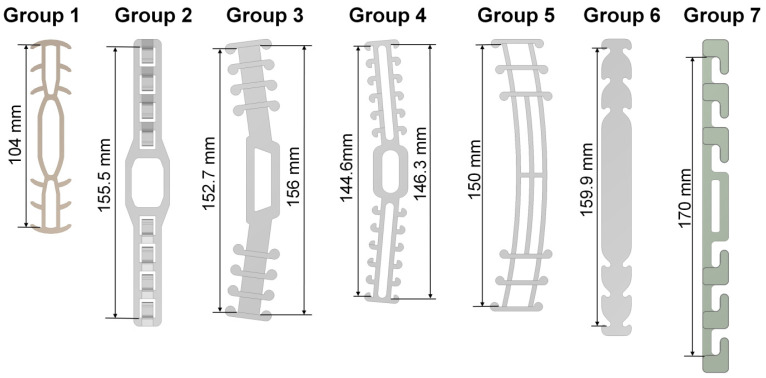
Seven different computer models of SMTRBs.

**Figure 2:**
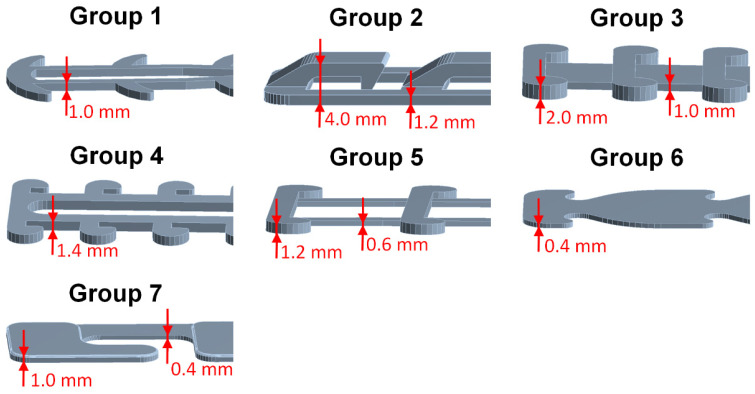
The relevant thicknesses of the SMTRB models for each group.

The purpose of this study is to evaluate the structural strength of different SMTRBs and to compare their wearing comfort. The bands studied were mostly those that were worn during the COVID-19 pandemic. The computer modeled those bands marked as being for “clinical use” on the NIH 3D Print Exchange website [16] of the National Institutes of Health of the U.S. Department of Health and Human Services. A designation for clinical use indicated that the design had been reviewed in a clinical setting and found to be suitable when manufactured using the specified printer type and material. Seven styles of SMTRB were studied (Figure 1). Their names were as follows: Group 1, ear savers for health workers; Group 2, surgical mask band for ear comfort—extra security V2; Group 3, surgical mask tension release band for ear comfort and extended use; Group 4, surgical mask tension release band STRISSE; Group 5, ear saver for surgical mask; Group 6, disposable ear relief strap; and Group 7, flexible mask hook. The relevant information for these SMTRBs is shown in Table 1. Figure 2 shows the relevant thickness data for these SMTRBs.

### 2.2. 3D-Printed SMTRBs

We first downloaded computer models of the seven SMTRBs from the NIH 3D Print Exchange website and imported the STL files for each model into a Material Extrusion 3D printer (C52, Henan Creatbot Technology Limited, Zhengzhou, Henan Province, China) for printing. The 3D printer material used in this study was poly lactic acid (PLA). The PLA material (PLA 3.0, Henan Creatbot Technology Limited, Zhengzhou, Henan Province, China) was produced by the original manufacturer. The line size of the material was 3.0 mm in diameter. The printer was set to a layer height of 0.2 mm, a fill density of 100%, a print speed of 40 mm/s, a nozzle temperature of 220 °C, and a bed temperature of 45 °C. According to previous research [21], when printing an object with a 3D printer, the structure of an object can have different strengths depending on the printing direction. However, the SMTRBs printed in this study were primarily used during the COVID-19 pandemic. Therefore, this study used the printing method that was the most time-saving manufacturing option. Table 2 shows the printing time of each band, as well as the weight and the length of the PLA material used. For each of the seven bands, ten copies were printed.

### 2.3. Stiffness Measurement for the SMTRB Structures

**Figure 3:**
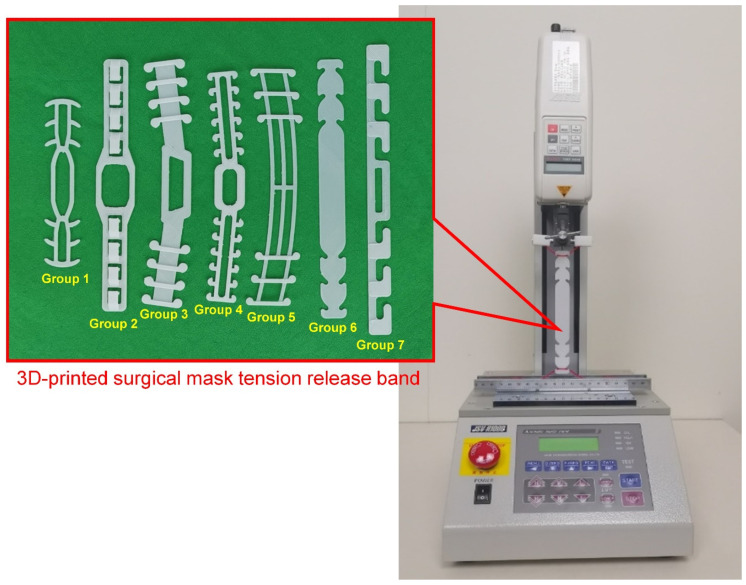
The solid model of a 3D-printed SMTRB is set up on the tensile testing machine.

Seven styles of 3D-printed SMTRB solid models (10 pieces in each group) were installed on a tensile testing machine (JSV-H1000, Japan Instrumentation System, Nara, Japan) (Figure 3). Nylon strings fixed on the tensile testing machine were hooked to both ends of each solid model. The nylon strings simulated a situation in which a person was wearing the band. A pre-force of 0.1 kgf was applied to both ends of the band, fixing it on the tensile testing machine. The tensile rate was 100 mm/min during the failure test. The tensile testing machine recorded the force loading and the tensile displacement during the stretching of the band.

### 2.4. Measurement of Pressure on the Face by a Sensor Array System

**Figure 4:**
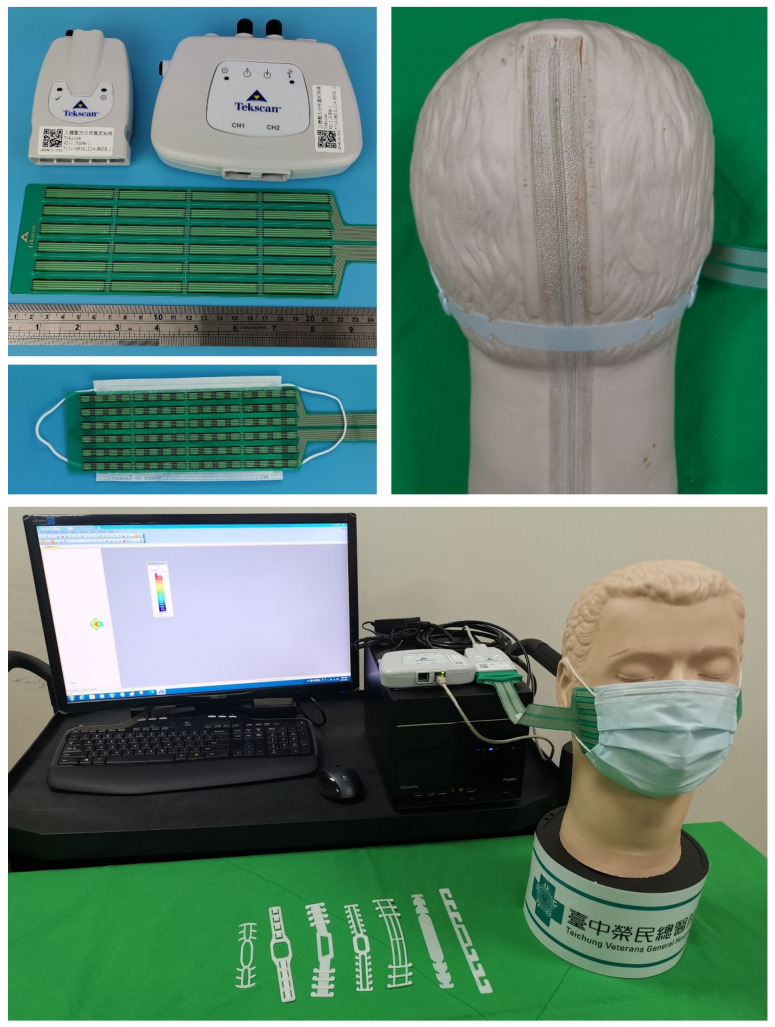
A mask was put on the dummy’s head with the sensor array measuring the pressure distribution on its face while using an SMTRB.

We used a pressure distribution sensor (Tekscan F-Socket sensor array 9811, Tekscan Inc., South Boston, MA, USA) fixed to the inside of a mask (mask size: 175 mm × 95 mm). The range of the pressure distribution sensor used in this study was 25 psi. The mask was mounted on a dummy head, and the sensor array was connected to the interceptor VersaTek Cuff. A 2-Port VersaTek Hub was then connected between the interceptor VersaTek Cuff and the computer. The pressure distribution on the dummy head (Laerdal Airway Management Trainer, Laerdal Medical, Stavanger, Norway) was measured by the sensor array when the mask was used together with the SMTRB (Figure 4). The head circumference of the dummy head was about 60 cm. Each style of SMTRB was measured ten times, and the maximum pressure values of the sensor were recorded. We also measured the pressure on the dummy face without an SMTRB.

### 2.5. Finite Element Analysis

The finite element analysis models employed in this study made use of the 3D printing computer model stl files. After importing the stl files into the 3D computer-aided design software ANSYS SpaceClaim (SCDM 18.0, ANSYS, Inc., Canonsburg, PA, USA), the computer models of the seven SMTRBs were also stored in igs files. Afterwards, the computer model igs files of each band were imported to the finite element analysis software ANSYS Workbench (version 18.0, ANSYS, Inc., Canonsburg, PA, USA) for analysis.

**Figure 5:**
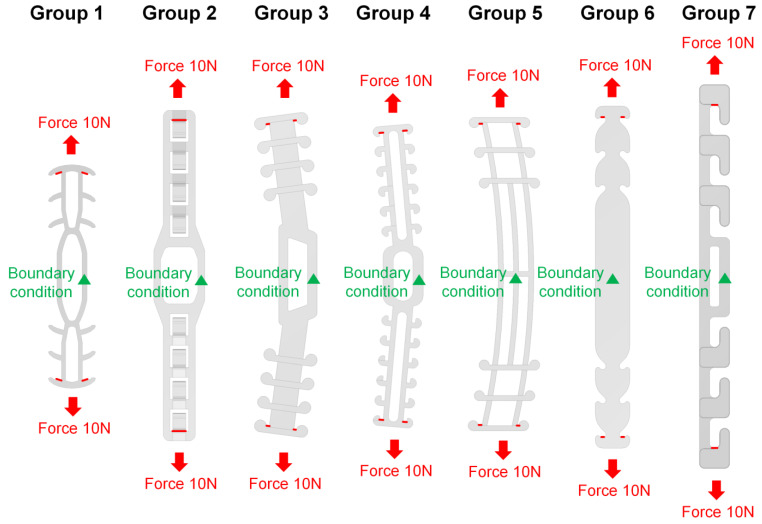
The boundary conditions and loading conditions for each group.

Since we studied different SMTRB structures under external force, boundary conditions and loading conditions were given (Figure 5). Because the bands were symmetrical structures, a boundary condition was given to the center position of the band (marked by the green triangle in Figure 5, with zero displacement of the X-, Y-, and Z-axes). Since the simulated band was affected by the mask ear lanyard, an axial external force of 10 N was provided [22]. The force positions on each band are marked by red symbols in Figure 5.

In terms of setting the material properties, we assumed the material was homogeneous, isotropic, and linearly elastic. According to a previous study, the Young’s modulus of PLA is 3149 MPa, with a Poisson’s ratio of 0.36 [23]. Tetrahedral meshes were the mesh elements used in our finite element analysis computer model. After the mesh had passed the convergence test, the model reached a 5% stop criterion of convergence. The finite element mesh model we used was reasonable for this band study. The control meshing size used in this study was 0.6 mm. Table 3 shows the number of nodes and elements in each group after meshing. The finite element analysis provided information, such as observation indicators regarding the stress distribution of von Mises stress on the SMTRBs.

## 3. Results

**Figure 6:**
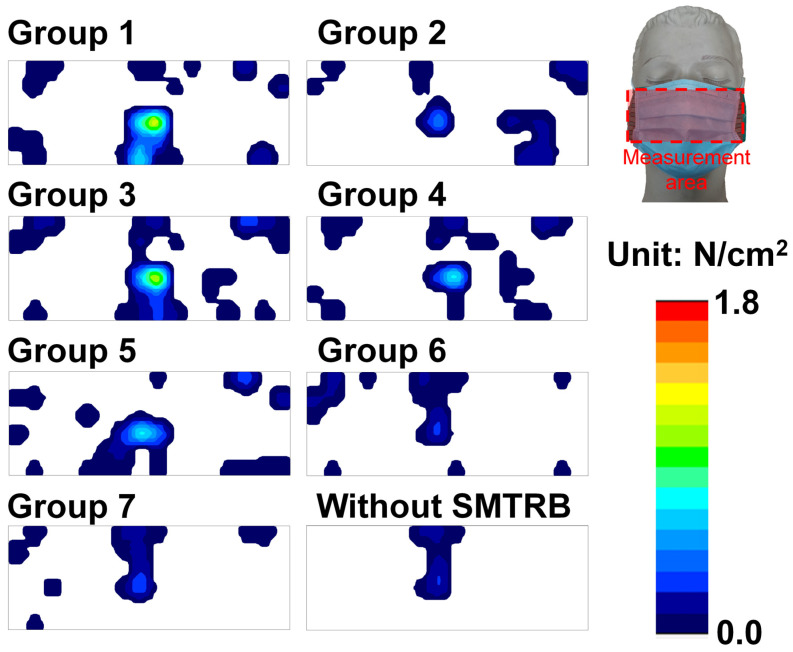
The pressure distribution on the dummy head when a mask was used with an SMTRB.

**Figure 7:**
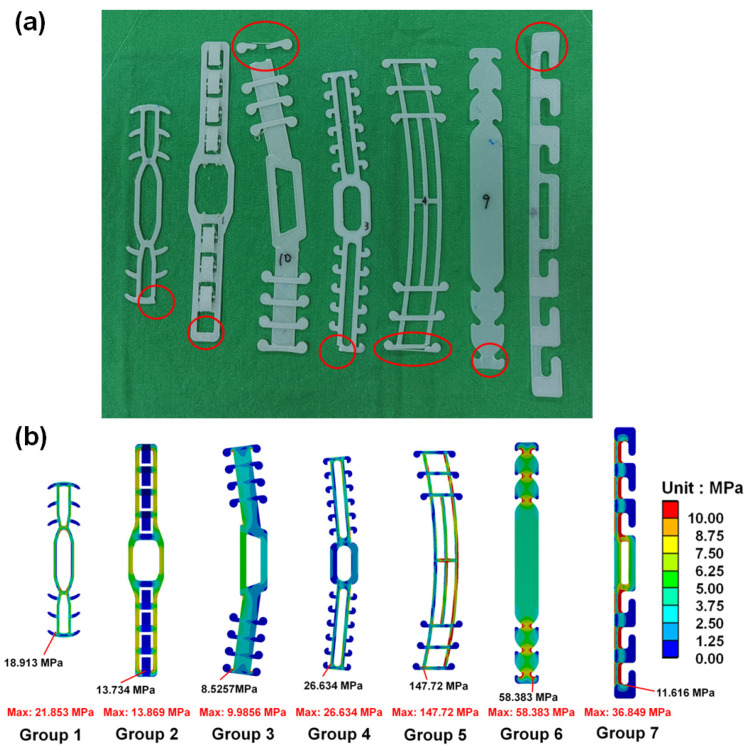
(**a**) The rupture of the SMTRBs in each group. (**b**) The distribution of von Mises stress on the surgical mask tension release bands in each group.

Table 4 shows the measurements gathered using the tensile testing machine and the sensor array system. We found that Group 3 withstood the greatest break force (14.379 ± 2.248 kgf), and Group 7 withstood the smallest force (1.756 ± 0.287 kgf). Regarding tensile displacement (tensile elongation) for broken bands, we found that Group 3 had the largest displacement (26.503 ± 5.264 mm), and Group 6 had the smallest displacement (6.304 ± 0.724 mm). In addition, the strain value (strain_1_ = 0.092433, strain_2_ = 0.068277, strain_3_ = 0.173563, strain_4_ = 0.106272, strain_5_ = 0.07962, strain_6_ = 0.039425, and strain_7_ = 0.030835) for each group was derived from the tensile displacement (calculated by the mean of each group) and the length of the SMTRB (calculated by the hooking position of each group of masks). Regarding the stiffness values, we found that Group 3 had the highest stiffness (0.616 ± 0.074 kgf/mm), and Group 7 had the lowest stiffness (0.387 ± 0.039 kgf/mm). In addition, we determined the pressure values for each group as measured by the sensor array system. We found that Group 3 had the highest pressure value (3.130 ± 0.305 N/cm^2^), and Group 7 had the lowest pressure value (0.901 ± 0.070 N/cm^2^). Figure 6 shows the pressure distribution on the dummy head when the mask was used with an SMTRB. The maximum pressure was almost always located at the nose tip. Figure 7a shows the failure or fracture of each group of bands as detected by the tensile testing machine. The red circle indicates where the band failed or fractured.

Next, we determined the distribution of von Mises stress for each group, as shown in Figure 7b. Note the maximum stress of each group of band and also the high-stress value of the ruptured area as revealed by the tensile testing machine. Results show that the high-stress value was the smallest in Group 3 and the largest in Group 5.

## 4. Discussion

From the results of the tensile testing machine in stretching the 3D-printed SMTRBs, we found that, for five groups (Group 1, Group 2, Group 3, Group 4, and Group 5), the bands most likely would have broken under a large force, near where the masks were in contact with the band (Figure 7a). In contrast, Group 6 and Group 7 bands would not have broken. The main reason was that the bands in Group 6 and Group 7 were relatively thin (about 0.4 mm), so almost all of them bent and slipped off.

Regarding the maximum external force that a band could withstand, we found that Group 3’s structural style withstood the largest tensile force, mainly due to its large thickness at the hooks where the masks were linked. Therefore, a larger force was required to break the band. The thickness at the hook of an SMTRB, therefore, affected its breaking force.

Regarding elongation and deformation, we found that except for Group 6 and Group 7, elongation values were between 5.242 and 6.304 mm as a consequence of slippage during testing. The elongation values of the bands in the other groups, after the bands were stretched to the breaking point, were between 9.613 mm and 26.53 mm. Regarding the strain capacity of the bands, we found that the structure of Group 3 had the highest strain capacity (0.173563). Regarding stiffness, we found that Group 3 and Group 4 had higher stiffness values, mainly due to their thicker bands. Since the hook of Group 4 was smaller than that of Group 3, it withstood less external force (7.832 kgf when destroyed by an external force). Therefore, the size and design of the hook of a band affected the amount of external force it could withstand.

With the sensor array system, we found that wearing the SMTRBs in Group 1 and Group 3 generated higher pressures. The Group 1 band generated more pressure because the axial length of the band was short. Consequently, the ear loops were more elongated before a successful hook-up with the band. The ear loops generated a large amount of tension in the mask; therefore, the face was subject to high pressure. Group 3′s finding was mainly due to the thickness structure of the band, which meant that it was not bent easily. The ear loops needed to be elongated to hook up with the band. Another observation for Group 4 was that, despite having a thicker band, it had a lightweight design with hollow structures. Thus, Group 4 bent better than Group 3, resulting in comparatively less pressure, or stress, on the face.

With greater pressure on the face, a mask fits better, but it is also less comfortable for the user. Therefore, using a longer SMTRB reduces the pressure and causes less discomfort. If a person does not use a longer band, their mask is not in good contact with the face. When wearing a mask, if a user adjusts the metal nose piece of the mask to bring the mask into closer contact with the nose, its protective function is enhanced [24]. Many commercially available masks have recently been improved in terms of their shape design to reduce the contact area between the mask and the face, thus reducing discomfort during mask wearing.

The results of the finite element analysis showed that when the SMTRBs were subjected to tensile force, high stress appeared in areas with small cross-sections, i.e., high stress at the band mask hooks, mainly due to a high stress concentration. According to previous studies [23], the yield strength of PLA material is 77 MPa, and the flexural strength is 88 MPa. For Group 5, with the tensile force at 10 N, the von Mises stress value of the hook position on the SMTRB exceeded those of the yield strength and flexural strength. Therefore, the Group 5 band likely breaks during wearing.

In summary, we found from our evaluations that each group of bands had advantages and disadvantages. Table 5 shows the advantages and disadvantages of each group of SMTRBs. Besides the evaluation of band structural strength during wearing, user comfort level is also important in situations with prolonged mask-wearing. Material loss and 3D printing time are also key points to be considered during the COVID-19 pandemic, when the demand for SMTRBs is high. The Group 1 SMTRB would be able to meet great demand from a hospital or institution if needed. Its fast printing speed and low cost would allow Group 1 SMTRBs to meet the demand in a short time. On the other hand, Group 5 SMTRBs performed significantly better than others. The Group 5 design is sturdy and requires a relatively short printing time, and the pressure on the face is relatively low while wearing. Therefore, the Group 5 SMTRB is also a good choice for regular use.

There are some limitations to this study. First, the masks we studied were commonly available on the market. We did not evaluate other masks with special designs. Second, in the tensile test, we used nylon string to simulate the ear loop material of a mask, mainly to avoid using the actual ear loops of a mask, because the ear loop material tends to stretch and elongate, affecting the results of attempts to measure the structural mechanics of the bands. Third, in the mechanical test, we only studied the outermost hook since the outer hook of the band is typically selected to hook onto the ear loops of a mask. Fourth, in the finite element analysis, we set homogeneous, isotropic, and linear elastic properties for the material, despite the different strengths of the 3D-printed models due to different positions during printing. Because we considered only the influence of the shape and structure of the SMTRBs, we set the materials to be homogeneous, isotropic, and with linear elasticity. Such a simplified setting avoided the problem of too many reference factors affecting the results.

This study mainly evaluated the structural strength, printing time, materials used, and the pressure applied to the face after wearing 3D-printed SMTRBs. Although each SMTRB has advantages and disadvantages, SMTRBs can reduce discomfort by avoiding the pressure exerted on the ears by ear loops under prolonged mask-wearing. Our results will provide not only evaluation methods for the mechanics and comfort of SMTRBs but also evaluation and design suggestions for the common SMTRB styles currently on the market. We hope that our results provide more design concepts and ideas for the manufacturers and designers of SMTRBs in order to produce optimized SMTRBs.

## 5. Conclusions

We used a tensile testing machine, a sensor array system, and finite element analysis to evaluate the structural strength and comfort of different styles of 3D-printed SMTRBs. We found that thinner SMTRBs were less likely to break but tended to slip off. Although thicker SMTRBs had strong structural strength, they caused masks to generate greater pressure on the face. Therefore, it is more desirable to have a lightweight design for SMTRBs so that they can be easily bent and to minimize mask pressure on the face. Finally, the hook size of SMTRBs also affected the magnitude of the external force. The findings of this study provide a biomechanical basis for designers of SMTRBs. In the future, a more durable and comfortable SMTRB can be designed based on the findings of this study.

## Figures and Tables

**Table 1:** Groups of SMTRBs.

SMTRB Information (Data Accessed on 29 July 2022)		
Group 1	File name	Ear Savers for Health Workers
	File website	https://3dprint.nih.gov/discover/3dpx-013860
	Thickness	1.0 mm
Group 2	File name	Surgical Mask Band for Ear Comfort—Extra Security V2
	File website	https://3dprint.nih.gov/discover/3dpx-013574
	Thickness	1.2 mm–4.0 mm
Group 3	File name	Surgical Mask Tension Release Band for Ear Comfort and Extended Use
	File website	https://3dprint.nih.gov/discover/3dpx-013410
	Thickness	1.0 mm–2.0 mm
Group 4	File name	Surgical Mask Tension Release Band STRISSE
	File website	https://3dprint.nih.gov/discover/3dpx-013615
	Thickness	1.4 mm
Group 5	File name	Ear Saver for Surgical Mask
	File website	https://3dprint.nih.gov/discover/3dpx-013675
	Thickness	0.6 mm–1.2 mm
Group 6	File name	Disposable Ear Relief Strap
	File website	https://3dprint.nih.gov/discover/3DPX-013564
	Thickness	0.4 mm
Group 7	File name	Flexible Mask Hook (Simple)
	File website	https://3dprint.nih.gov/discover/3dpx-013752
	Thickness	0.4 mm–1.0 mm

**Table 2:** The weight and length of the PLA material used, as well as the printing time for each SMTRB.

	Group 1	Group 2	Group 3	Group 4	Group 5	Group 6	Group 7
Printing Time (min)	7	35	31	21	13	13	15
The Weight of the PLA (g)	1	4	4	3	2	2	2
The Length of the PLA (m)	0.12	0.49	0.49	0.3	0.19	0.23	0.25

**Table 3:** The number of nodes and elements after meshing in each group.

Mesh	Group 1	Group 2	Group 3	Group 4	Group 5	Group 6	Group 7
Number of Nodes	29,469	77,388	69,230	58,450	45,118	58,341	79,394
Number of Elements	14,566	41,403	35,567	29,091	21,732	28,345	41,328

**Table 4:** The maximum break force, the tensile displacement of the SMTRB, and the stiffness as measured by the tensile testing machine. The pressure measured by the sensor array system is also displayed.

Observed Index		Group 1	Group 2	Group 3	Group 4	Group 5	Group 6	Group 7	Without SMTRB
Maximum Break Force (kgf)	Max	4.840	6.540	17.890	8.780	7.740	3.650	2.120	-
	Mean	3.687	5.322	14.379	7.832	5.247	2.828	1.756	-
	SD	0.854	0.606	2.248	0.661	1.080	0.452	0.287	-
Tensile Displacement (mm)	Max	11.060	13.530	33.190	19.300	17.520	7.350	7.400	-
	Mean	9.613	10.617	26.503	15.367	11.943	6.304	5.242	-
	SD	1.157	1.493	5.264	1.607	2.449	0.724	0.807	-
Stiffness (kgf/mm)	Max	0.546	0.647	0.751	0.723	0.590	0.588	0.427	-
	Mean	0.448	0.588	0.616	0.614	0.507	0.532	0.387	-
	SD	0.077	0.035	0.074	0.086	0.046	0.051	0.039	-
Pressure (N/cm^2^)	Max	6.090	1.880	3.648	1.543	2.518	1.543	0.990	0.726
	Mean	4.917	1.556	3.130	2.662	2.097	1.300	0.901	0.515
	SD	0.639	0.258	0.305	0.478	0.224	0.171	0.070	0.147

**Table 5:** The advantages and disadvantages of the SMTRBs in each group.

Group	Advantages	Disadvantages
Group 1	The shortest 3D printing time and the saving of 3D printing material.	Greater pressure on the face when wearing.
Group 2	The hook structure design prevents the ear loops from falling off.	Longer 3D printing time. Higher cost of 3D printing material. Fragile hook design.
Group 3	Stronger structural strength.	Longer 3D printing time. Higher cost of 3D printing material. Greater pressure on the face.
Group 4	Stronger structural strength and bending ability.	The hook is too small to hold.
Group 5	Relatively shorter 3D printing time and lower cost of material.	The stress value of the hook position is large and easy to break.
Group 6	Relatively shorter 3D printing time. Lower cost of 3D printing material. Structural design is not easy to damage.	The ear loops slip off easily when the mask is hooked.
Group 7	Relatively shorter 3D printing time. Lower cost of 3D printing material. Structural design is not easy to damage.	The ear loops slip off easily when the mask is hooked.

## Data Availability

Not applicable.
